# UV-A Radiation: Safe Human Exposure and Antibacterial Activity

**DOI:** 10.3390/ijms24098331

**Published:** 2023-05-05

**Authors:** Angela Sandri, Aldo Tessari, Danilo Giannetti, Alberto Cetti, Maria M. Lleo, Federico Boschi

**Affiliations:** 1 Department of Diagnostics and Public Health, University of Verona, Strada Le Grazie 8, 37134 Verona, Italy; angela.sandri@univr.it (A.S.); maria.lleo@univr.it (M.M.L.); 2 FOR ALL Srl, Via 8 Marzo 10-12, Bussolengo, 37012 Verona, Italy; aldo.tessari@for-all.it (A.T.); alberto.cetti@for-all.it (A.C.); 3 Agilux Srl, Via Augusto Anfossi 19, 20135 Milan, Italy; danilo.giannetti@agilux.it; 4 Department of Engineering for Innovation Medicine, University of Verona, Strada Le Grazie 8, 37134 Verona, Italy

**Keywords:** UV-A radiation, skin exposure, eye exposure, *Escherichia coli*, *Pseudomonas aeruginosa*, *Staphylococcus aureus*

## Abstract

UV radiation is used for sterilization but has adverse health effects in humans. UV-A radiation has lower antimicrobial effect than UV-B and UV-C but constitutes a lower health risk, opening up the possibility to sanitize environments with human presence in controlled exposure conditions. We investigated this possibility by identifying safe exposure conditions to a UV-A lamp along with efficient sanitization of the environment. The human exposure limits were calculated following the guidelines provided by the International Commission on Non-Ionizing Radiation Protection and the International Commission on Illumination. Antibacterial activity was evaluated on *Escherichia coli*, *Pseudomonas aeruginosa* and *Staphylococcus aureus*. The maximum human exposure duration has been identified at different irradiation distance and angle, increasing with the increase of both parameters. Bactericidal activity was observed in all microorganisms and was higher with higher exposure time and at lower distance from the source. Noteworthily, in equal conditions of radiant exposure, the exposure time impacts on the bactericidal activity more than the distance from the source. The modulation of factors such as distance from the source, exposure time and irradiation angle can enable effective antibacterial activity and human safety. Prolonged direct irradiation of the surfaces associated with indirect human exposure represents the condition of greater efficacy and safety.

## 1. Introduction

The antimicrobial activity of UV radiation has been known for long time and is used in applications such as sterilization [1,2,3,4,5,6,7,8]. The efficiency of UV antimicrobial effects mostly depends on the wavelength range. Based on it, UV light is categorized into three bands: UV-A (315 to 400 nm), UV-B (280 to 315 nm), and UV-C (100 to 280 nm). It is recognized that the UV-C radiation has the most powerful antimicrobial effect, as it directly damages the integrity of microbial DNA. UV-B acts using the same mechanism, at a smaller extent. In contrast, UV-A (and UV-B, to some degree) generates reactive oxygen species (ROS) that can damage multiple cellular targets, including proteins, lipids, and nucleic acids, and can kill the microorganisms [2,3].

However, the UV radiation also has adverse health effects for the eye and skin in humans, as these are the surfaces of the body directly exposed to the radiation. Exposure of the skin to UV results in inflammation (erythema/sunburn), whose extent depends on the skin phototype, and is the main environmental factor contributing to development of skin cancer like basal cell carcinoma, squamous cell carcinoma and, more rarely, melanoma [9]. Exposure of the eyes to UV may cause damage to the corneal epithelium (photokeratitis) and contribute to cataracts formation [10]. UV-C and UV-B cause DNA damage, the most common being the formation of cyclo-butane pyrimidine dimers and pyrimidine-pyrimidone photoproducts [11,12]. If not repaired, these mutagenic lesions can initiate carcinogenesis [13]. Although there are very few studies that have investigated UV-C adverse health effects in humans, UV-C is considered carcinogenic due to its mode of action and induced DNA damage similar to UV-B [14]. Moreover, UV-B and UV-A induce oxidative stress, which can indirectly contribute to DNA damage and, thus, skin cancer [15].

Although UV-A radiation has a lower antimicrobial effect, at the same time it constitutes a lower health risk thanks to the absence of direct effects (like those caused by UV-B and UV-C) [16]. This opens up the possibility that UV-A lamps could be used to sanitize environments where people are present, in controlled exposure conditions, e.g., where the human presence is time-restricted, or where people are not directly exposed to the UV-A radiation. The aim of this study is to investigate this possibility by identifying the exposure conditions that could enable safety for humans (i.e., exposure does not cause harm) and efficient sanitization of the environmental surfaces (i.e., antibacterial effect on exposed surfaces).

## 2. Results

### 2.1. Exposure Limits at Maximum Irradiance

**Figure 1:**
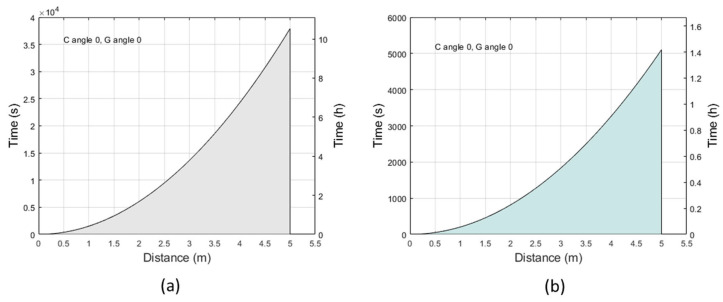
Exposure limits to the UV-A radiation perpendicular to the source (angles C and G = 0°). (**a**) The curve indicates the time and distance conditions at which the maximum effective radiant exposure (30 J/m^2^) is reached for the eyes and skin to UV radiation; the area below the curve indicates allowed exposure times and distances, at which the effective radiant exposure is less than 30 J/m^2^. (**b**) The curve indicates the time and distance conditions at which the maximum absolute ocular radiant exposure (10,000 J/m^2^) to UV-A radiation is reached; the area below the curve indicates allowed exposure times and distances, at which the maximum absolute radiant exposure is less than 10,000 J/m^2^.

The maximum duration of exposure in relation to the distance from the source was calculated using the exposure limits provided by the International Commission on Non-Ionizing Radiation Protection (ICNIRP) guidelines [17]. Figure 1 shows the maximum effective UV radiant exposure for eye and skin (Figure 1a) and the maximum absolute UV-A ocular radiant exposure (Figure 1b) in conditions of maximum irradiance (C and G = 0°; Figure S1). In both graphs, the area below the curve indicates the allowed exposure times (within the ICNIRP limit) in relation to the distance from the source. The maximum duration of exposure increases as the distance from the source increases.

### 2.2. Radiant Exposure Limits and Irradiation Angles

**Figure 2:**
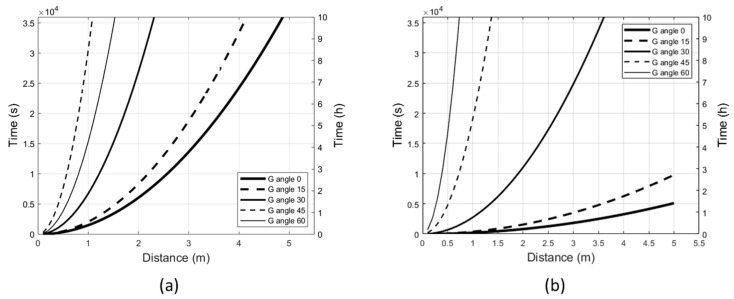
Limits of exposure to UV-A radiation at different angles of irradiation (G = 0°, 15°, 30°, 45°, 60°; C = 0°). (**a**) The curves indicate the time and distance at which the maximum effective radiant exposure (30 J/m^2^) is reached for the eye and skin with varying G angle. (**b**) The curves indicate the time and distance at which the maximum absolute radiant exposure (10,000 J/m^2^) is reached for the eye with varying G angle. In both figures, the areas below the curves (not shown) correspond to times and distances of permitted exposure.

The radiant exposure limits to the UV-A lamp vary in relation to the irradiation angle. At a fixed distance from the lamp, the permitted exposure duration increases with increase of the G angle. Figure 2 shows the maximum effective UV radiant exposure for eye and skin (Figure 2a) and the maximum absolute UV-A ocular radiant exposure (Figure 2b) at different irradiation angles, e.g., at 3 m from the source, the exposure limit of the eye to the perpendicular irradiation (G = 0°) is reached in less than 1 h (Figure 2b); by tilting the lamp with angle G = 30°, the exposure limit is reached in approximately 7 h.

### 2.3. Erythemal Exposure Limits at Maximum Irradiance and Varying Irradiation Angles

**Figure 3:**
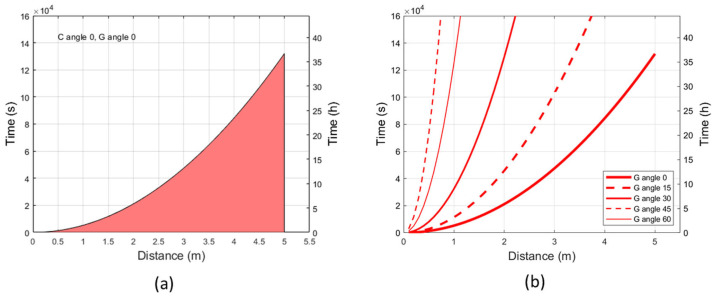
Erythemal exposure limits to the UV-A radiation. (**a**) The curve indicates the time and distance conditions at which the erythemal effective radiant exposure of 2 SED is reached with to UV-A radiation perpendicular to the source (angles C and G = 0°); the area below the curve indicates allowed exposure times and distances at which the erythemal radiation is <2 SED. (**b**) The curves indicate the time and distance at which the erythemal effective radiant exposure of 2 SED is reached at different angles of irradiation (G = 0°, 15°, 30°, 45°, 60°; C = 0°).

In addition to radiant exposure limits set by the ICNIRP guidelines [15], the erythemal exposure limits were calculated following the guidelines provided by the International Commission on Illumination [18]. Figure 3a shows the level of erythemal effective radiant exposure which may produce just minimal erythema in the most sensitive skin phototype (phototype I on Fitzpatrick scale [9]), corresponding to a Standard Erythema Dose (SED) of 2, in conditions of maximum irradiance (C and G = 0°; Figure S1). The area below the curve indicates the allowed exposure times (within the exposure limit of 2 SED) in relation to the distance from the source. The maximum duration of exposure increases as the distance from the source increases. Figure 3b shows the erythemal effective radiant exposure of 2 SED at different irradiation angles. At a fixed distance from the lamp, the erythemal exposure limits increase with increase of the G angle, e.g., at 1 m from the source, the erythemal exposure limit for the skin phototype I to the perpendicular irradiation (G = 0°) is reached in 90 min; by tilting the lamp with angle G = 30°, the exposure limit is reached in around 9 h. The individual erythemal exposure limit per phototype was also calculated at 1 m from the source in conditions of maximum irradiance (C and G = 0°), increasing with skin phototype as shown in Table 1.

### 2.4. Antibacterial Activity over Exposure Time and Irradiation Distance

**Figure 4:**
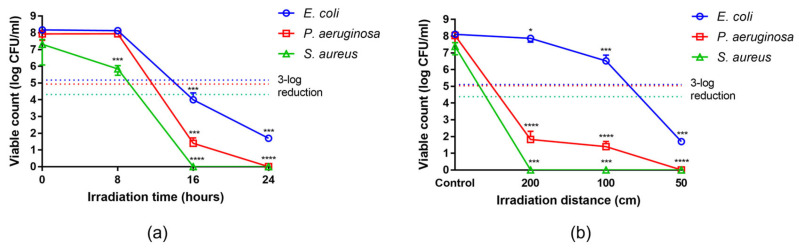
Viable count at varying exposure times and irradiation distances. (**a**) Viable count (log CFU/mL) at 8, 16, and 24 h at 50 cm fixed distance from the UV-A source, and (**b**) at 200, 100, 50 cm distance for 24 h. Viable count is expressed as log CFU/mL. Each value represents the mean ± SD of 6 drops (2 per plate). The cut-off for bactericidal activity (3-log reduction) is indicated for each bacterial species (dotted line). Statistical analysis of irradiated vs control plates was performed by Kruskal-Wallis test followed by Dunn’s multiple comparisons test; * *p* < 0.05, *** *p* < 0.001, **** *p* < 0.0001.

Antibacterial activity was assessed on *Escherichia coli*, *Pseudomonas aeruginosa* and *Staphylococcus aureus* in conditions of maximum irradiance (C and G = 0°; Figure S1) at different exposure times (8, 16, and 24 h) and was observed to increase with increase of the exposure duration. In all bacterial species evaluated, a bactericidal effect (defined as a reduction in Colony Forming Units (CFU) of at least 3-log or 99.9% in irradiated vs. control plates) was observed after 16 and 24 h of exposure, causing a significant reduction in the viable count. The irradiation was particularly effective against *P. aeruginosa* and *S. aureus*, causing a reduction of 6-log and 7-log, respectively, already at 16 h. In contrast, a 6-log reduction for *E. coli* was observed at 24 h (Figure 4a, Table S1).

Effects of the irradiation distance on antibacterial activity were observed at 50, 100, and 200 cm. In all bacterial species evaluated, bactericidal activity was observed to increase with decrease of the distance from the source. The viable count of *P. aeruginosa* and *S. aureus* was significantly reduced by 6-log and 7-log respectively already at 200 cm, while *E. coli* showed a comparable reduction (6-log) only at 50 cm (Figure 4b, Table S2).

As a qualitative observation, after 24 h of exposure *E. coli* colonies were visible in the control plates but not in the plates exposed to the UV-A radiation. In the latter, the colonies became visible after further 24 h of incubation at 37 °C, suggesting a bacteriostatic activity of the radiation on this microorganism. This effect was observed at distances of 100 and 200 cm from the UV-A source.

### 2.5. Bactericidal Activity in Relation to the Irradiance of the Source

**Figure 5:**
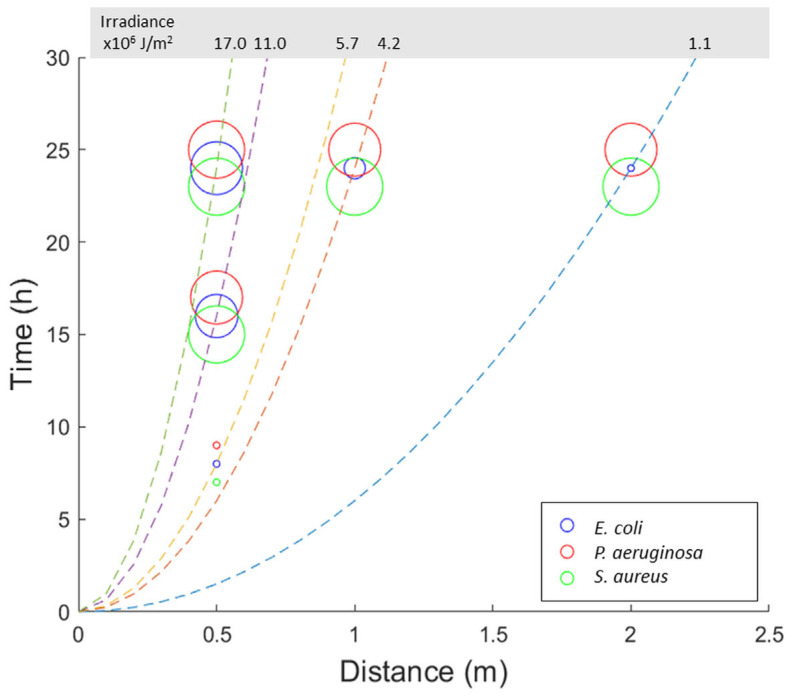
Bactericidal activity in relation to the radiant exposure of the UV-A radiation. The dotted lines indicate the absolute irradiance of UV-A radiation calculated in relation to the duration of exposure and the distance from the source. The circles indicate the bactericidal activity observed at certain times and distances of exposure on the different bacterial species, distinguished by color; the larger the size of the circle, the greater the bactericidal activity (log reduction) observed.

The absolute irradiance of the UV-A radiation emitted by the lamp (at angles C and G = 0°) was calculated in relation to the time and distance of exposure of the bacterial populations. Figure 5 shows the bactericidal activity of the UV-A lamp on the different bacterial species in relation to the absolute irradiance at varying times and distances of exposure. For two of the microorganisms tested (*P. aeruginosa* and *S. aureus*), the bactericidal activity is greater in the experimental condition with the lowest radiant exposure (24 h of exposure and 2 m of distance from the source) compared to an experimental condition having a radiant exposure greater than about 5 times (50 cm, 8 h). An analogous situation, to a smaller extent, is observed for *E. coli* too. The bactericidal activity of the lamp therefore does not depend solely on irradiance but, with equal radiant exposure, it is greater as the exposure time increases, even in the event of an increase in the distance from the source. 

## 3. Discussion

Microbial contaminants are transmitted in the environment both by means of aerosol (the droplets emitted during normal breathing and when coughing or sneezing) and through the objects people touch [19,20,21]. Since COVID-19 pandemics, the search for systems that can help contain and eliminate microbial agents to prevent contamination is more intense than ever [22]. The current needs are to constantly maintain a high standard of sanitization, but this entails more frequent interventions by cleaning services and higher costs. It is therefore necessary to evaluate new systems able to assist and support the sanitization of environments, in particular of those with a high human traffic (e.g., public restrooms, transportation hubs such as airports and stations, public offices, etc.).

The recent advent on the market of LEDs with UV emission has made it possible to have stable, durable, and significantly more compact UV light sources that could support sanitization thanks to their known antimicrobial effects [23]. However, UV radiation also poses serious risks for human health, thus the irradiation conditions need to be carefully evaluated to sanitize the environments without causing harm [24]. UV-A has the lowest bactericidal effect among UV bands, but it also is the least dangerous for humans, as it only causes indirect damages through oxidative stress mechanisms [24]. Therefore, UV-A is the most likely to be suitable for use in environments where people are present, but appropriate conditions must be identified to maximize sanitization while minimizing health risks.

In the present study, we examined the most appropriate irradiation conditions for a UV-A lamp containing 28 LED diodes. The exposure duration limits for humans, within which the emitted radiation does not cause biological damage to the eye and skin according to the ICNIRP guidelines [17] nor erythema even in the most sensitive skin phototype according to CIE guidelines [18], vary according to the distance from the source and to its irradiation angle. At distances compatible with indoor lighting, the inclination of the source with angle G ≥ 30° allows exposure for several hours without incurring in skin and eye problems. In general, the exposure duration limit increases with increase of the distance from the source, and with increase of the irradiation angle. Conversely to the latter trend, the exposure duration limit of eye and skin in terms of maximum effective radiant exposure, as well as the erythemal exposure limit, was higher with G angle = 45° than with G angle = 60°. However, it must be considered that irradiance at these angles is extremely low, comparable to background noise. 

The most stringent exposure limit concerned the eye rather than the skin, meaning that the absolute ocular radiant exposure limit is reached faster than the effective radiant exposure limit (for eye and skin) and the erythemal exposure limit. It is worth to note that the ICNIRP’s exposure limits were quantified in terms of time-weighted averaged radiant exposure and reflect the maximum average exposure a worker can be subjected to without experiencing adverse health effects over a standardized 8-h work period. However, the eyes (and to a lesser extent the skin) are anatomically protected against UV exposure from overhead sources. e.g., the radiation coming from above is perpendicular to the optical axis of a subject in an upright position and do not directly affect the eyes; moreover, the continuous movement of the eyelids create interruptions in the exposure (discontinuous). As such, although our study lacks real exposure data, which cannot be obtained in a laboratory setting, the exposure duration limits here calculated can be considered conservative with respect to the real environmental conditions.

The UV-A lamp showed bactericidal activity against various bacterial species frequently present in the environment and which, in particular conditions, can cause infections in humans (opportunistic pathogens). Direct exposure to the radiation elicited bactericidal effects on *E. coli*, *P. aeruginosa* and *S. aureus*, representing classes of bacteria with different characteristics (Gram-negative/Gram-positive). *S. aureus* was the microorganism most sensitive to the bactericidal effects of the UV-A radiation, while *E. coli* was the least sensitive. Microbial sensitivity to UV is known to vary widely not only between different bacteria, but even between different strains belonging to the same species [25,26,27,28]. It is important to note that the *E. coli* ATCC 8739 strain used in our study has previously been reported to be less sensitive to UV-C light than other *E. coli* strains (e.g., O157:H7) [25], supporting its low sensitivity to the UV-A radiation.

Noteworthily, we could also observe a bacteriostatic effect of the UV-A radiation against *E. coli*, likely due to the induction of oxidative cell damage by UV-A, which contributes to microbial inactivation [29,30,31]. The DNA repair system has been reported to be triggered at a low fluence rate in *E. coli*, suggesting that the growth delay could also occur to allow DNA repair before cells begin to divide [32]. Conversely, in *P. aeruginosa* the UV-A radiation has been suggested to also cause indirect DNA damage due to the well-known deficiencies in the DNA repair systems of this microorganism [33]. This could explain why *P. aeruginosa* is more sensitive to UV-A radiation than *E. coli*.

In conclusion, effective bactericidal or bacteriostatic activity can be obtained through prolonged exposure to UV-A over time, even at long distances. Although this may not be sufficient to sterilize environments where microbes are constantly added up, like public spaces with high human traffic, it may nonetheless help containing the microbial load. The modulation of factors such as distance from the source, exposure time and irradiation angle can make it possible to reduce or contain the bacterial load present on the surfaces exposed to radiation without causing biological damage to humans. Prolonged direct irradiation of the surfaces associated with human exposure to indirect light represent the condition of greater efficacy and safety.

## 4. Materials and Methods

### 4.1. UV-A Source

**Figure 6:**
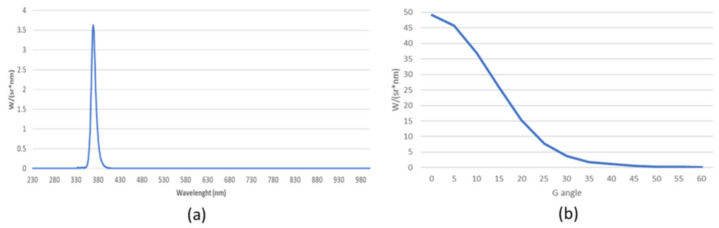
UV-A lamp irradiance in the wavelength region 230–1000 nm (**a**) perpendicularly to the source (angles C and G = 0°) and (**b**) at different angles with respect to the source (G from 0° to 60°, and C = 0°).

The UV-A source is a lamp (FOR ALL, Verona, Italy) equipped with n = 28 UV-A (λ = 365 nm) LED diodes (Seoul Viosys, Ansan-si, South Korea) and wide beam optics (Khatod, Milan, Italy) specific for UV applications. The irradiance of the UV-A lamp was measured at wavelengths between 230 and 1000 nm (Figure 6a) and at different angles (in far-field conditions) at a certified testing laboratory (Zanelli Srl, Vailate, Cremona, Italy). The lamp has an emission between 360 and 400 nm (UV-A spectrum), reaching the maximum irradiance at 370 nm. The irradiance is at its maximum when the radiation is perpendicular to the source (angles C and G = 0°) and decreases as the angle G varies (Figure 6b). G is the angle in the plane perpendicular to the lamp axis, C is the angle measured in the plane passing through the lamp with G = 0° (Figure S1).

### 4.2. Exposure Limits

The exposure limits to the UV-A lamp have been calculated following the guidelines provided by the International Commission on Non-Ionizing Radiation Protection (ICNIRP) on exposure limits to UV radiation [17]. According to these guidelines, the exposure of eyes and skin (unprotected) to UV radiation (180–400 nm) must not exceed the effective radiant exposure limit (relating to the biological effect) of 30 J/m**^2^** within 8 h. In addition, ocular exposure to UV-A radiation (315–400 nm) must not exceed the absolute radiant exposure limit of 10,000 J/m**^2^** within 8 h. Irradiance values between 230 and 400 nm were used to calculate the UV-A lamp exposure limits. The effective irradiance was calculated using the parameter S (λ), i.e., the spectral efficacy relative to a reference wavelength for which the biological damage is maximum [17]. 

The erythemal exposure limits were calculated following the guidelines provided by the International Commission on Illumination (CIE) [18]. According to these guidelines, the unit of erythemal radiation is the Standard Erythema Dose (SED), where 1 SED is equivalent to an erythemal effective radiant exposure of 100 J/m^2^, and it requires an exposure of 2 SED to produce just minimal erythema in the skin phototype I, the most sensitive to UV radiation [9]. Irradiance values between 250 and 400 nm were used to calculate the UV-A lamp erythemal exposure limits. The erythemal effective irradiance was calculated using the erythema action spectrum, i.e., the relative effectiveness of different wavelengths of UV radiation to cause erythema in human skin 8–24 h after exposure [9].

### 4.3. Antibacterial Activity

Bactericidal activity of the UV-A lamp was evaluated on *Escherichia coli* (ATCC 8739), *Pseudomonas aeruginosa* (PAO1 strain, ATCC 47085) and *Staphylococcus aureus* (strain SR2014). Starting from a 0.5 McFarland bacterial suspension in 0.45% saline solution, six serial dilutions 1:10 were prepared and 20 μL of each dilution were deposited twice on an LB (Luria Bertani) agar plate (Figure S2). The test plates were closed with polystyrene lids (with low absorbance in the UV-A spectrum [34]) and exposed to the UV-A radiation (perpendicularly below the source, i.e., C = 0°, G = 0°). The control plates were closed with polystyrene lids and covered with aluminum foil. 

To study the effect of time, plates were exposed for 8, 16, or 24 h at 50 cm distance from the UV-A source. To study the effect of the distance, plates were exposed at 50, 100 or 200 cm distance from the UV-A source for 24 h. Each condition was repeated in triplicate. After irradiation, plates were incubated at 37 °C for 24–48 h (without irradiation), and the colony forming units (CFU) were counted. Reduction in viable count was calculated as log10 (CFU∙mL^−1^ in control plates/CFU∙mL^−1^ in irradiated plates). Bactericidal effect was defined as a CFU reduction ≥3 log. Statistical analysis of CFU reduction in irradiated vs control plates was performed by Kruskal-Wallis test followed by Dunn’s multiple comparisons test. Bacteriostatic effect was qualitatively assessed when CFU were visible right after UV-A irradiation in control plates only and became visible in irradiated plates too after the 24–48 h incubation (without irradiation) at 37 °C. 

## Figures and Tables

**Table 1:** Erythemal exposure limits to the UV-A radiation per skin phototype. Fitzpatrick skin phototypes, sunburn susceptibility, minimal erythemal radiation [9] and exposure limit at 1 m from the UV-A lamp with maximum irradiance (C and G = 0°) are indicated.

Skin Phototype	Sunburn Susceptibility	Minimal Erythemal Radiation (SED)	Erythemal Exposure Limit (min)
I	Very high	2	90
II	High	3	135
III	Moderate	4	180
IV	Low	5	225
V	Very low	7	315
VI	Extremely low	≥9	≥405

## Data Availability

Data are available from the corresponding author on reasonable request.

## Supplementary Materials

The following supporting information can be downloaded at: https://www.mdpi.com/article/10.3390/ijms24098331/s1.
