# Supplementary material for: UV-A Radiation: Safe Human Exposure and Antibacterial Activity
Source: Int J Mol Sci. 2023 May 5;24(9):8331. doi: 10.3390/ijms24098331 (PMC10179708; doi:10.3390/ijms24098331)
Supplement: Supplementary file 1 [file ijms-24-08331-s001.zip › ijms-2303082-supplementary.pdf]

Supplementary Materials

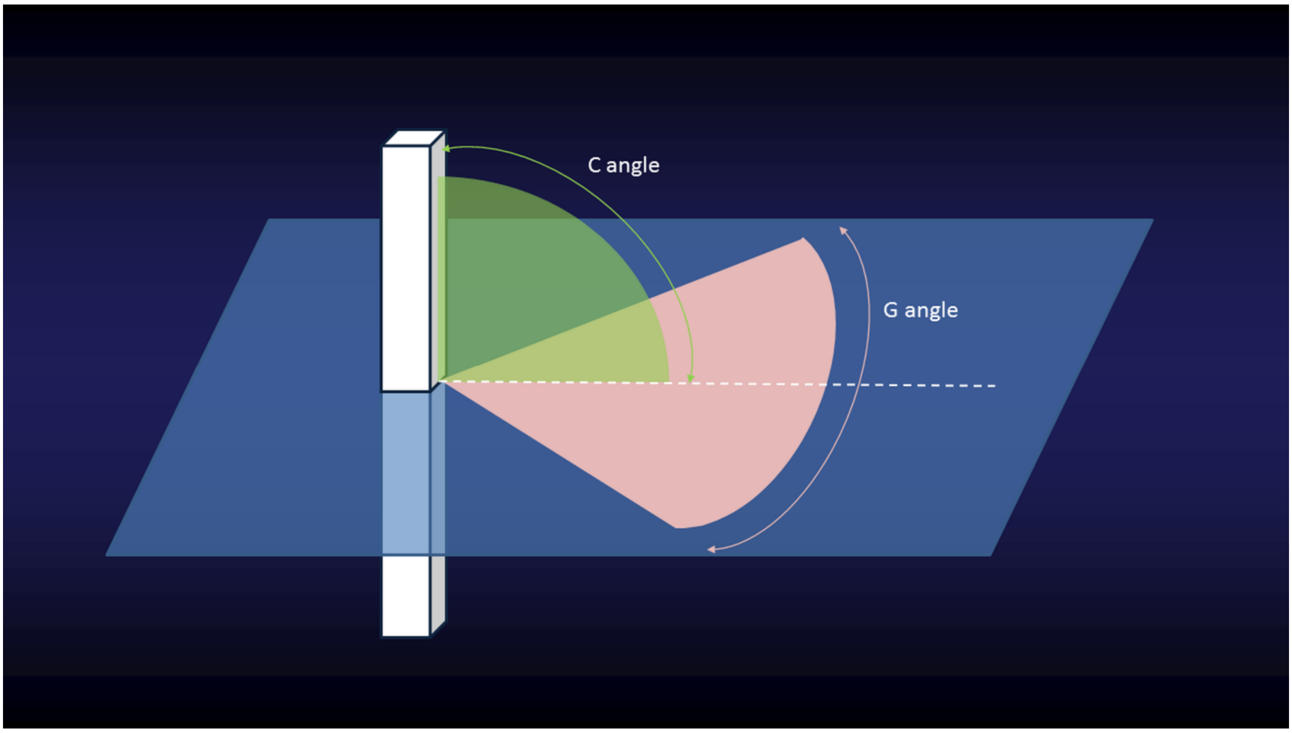

Figure S1. Graphical representantion of G and C angles with respect to the lamp (white parallelepiped).

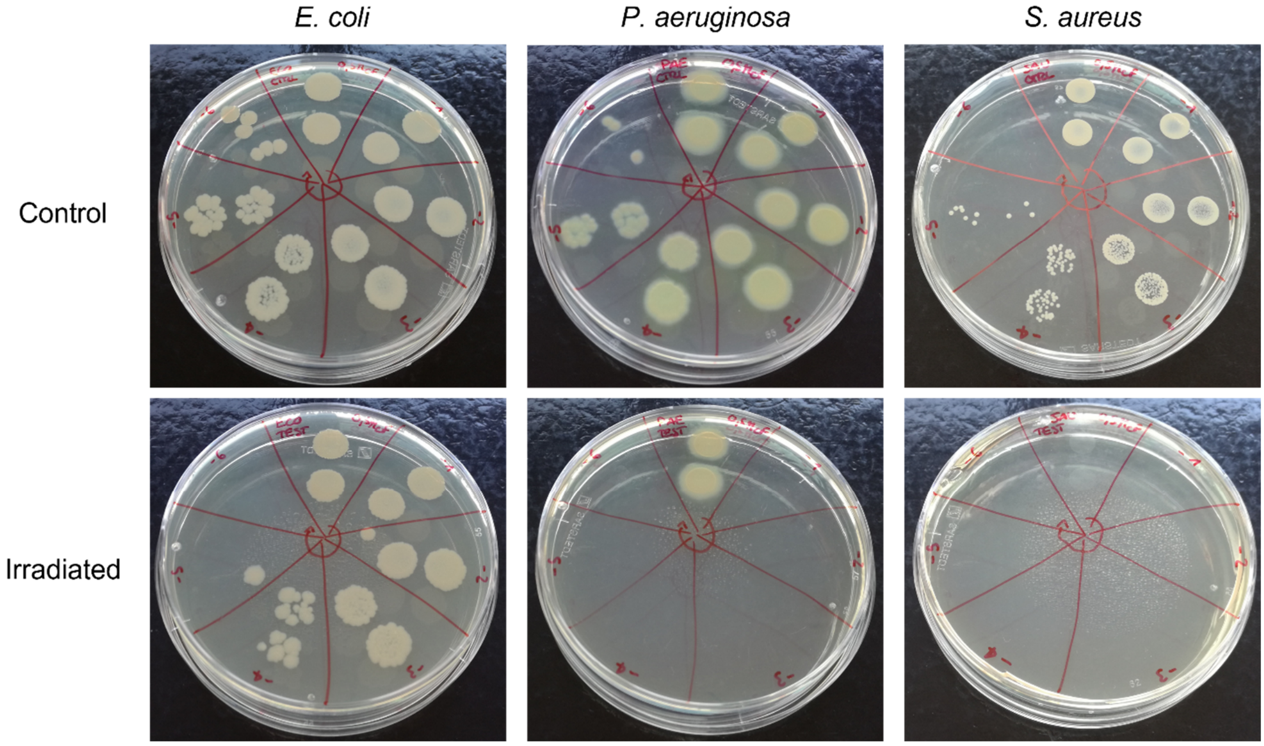

Figure S2. Representative pictures of plates irradiated for 24 hours at 1 meter distance from the UV-A source and control plates. Plates were divided in sections containing decreasing dilutions of bacterial suspensions (clockwise); each section contains 2 drops (20 µl each) of the appropriate dilution.

Table S1. Reduction of bacterial populations at 50 cm distance from the UV-A source.

**Reduction**	**8h**		**16h**		**24h**	
	**%**	**log**	**%**	**log**	**%**	**log**
E. coli	7.95%	0	99.99%	4	99.9999%	6
P. aeruginosa	19.70%	0	99.9999%	6	>99.9999%	7
S. aureus	96.98%	1	>99.9999%	7	>99.9999%	7

Table S2. Reduction of bacterial populations after 24 hours UV-A irradiation.

**Reduction**	**50 cm**		**100 cm**		**200 cm**	
	**%**	**log**	**%**	**log**	**%**	**log**
E. coli	99.9999%	6	97.06%	1	10.95%	0
P. aeruginosa	>99.9999%	7	>99.9999%	6	>99.9999%	6
S. aureus	>99.9999%	7	>99.9999%	7	>99.9999%	7
